# LIMK1 Interacts with STK25 to Regulate EMT and Promote the Proliferation and Metastasis of Colorectal Cancer

**DOI:** 10.1155/2022/3963883

**Published:** 2022-02-28

**Authors:** Xuecheng Sun, Shaotang Li, Han Lin

**Affiliations:** ^1^Department of Gastroenterology, The First Affiliated Hospital of Wenzhou Medical University, Wenzhou, Zhejiang, China; ^2^Department of Colorectal Surgery, The First Affiliated Hospital of Wenzhou Medical University, Wenzhou, Zhejiang, China; ^3^Department of Central Laboratory, The First Affiliated Hospital of Wenzhou Medical University, Wenzhou, Zhejiang, China

## Abstract

**Objective:**

To investigate the interaction between LIMK1 and STK25 and its expression in colon cancer and its effect on the malignant evolution of colon cancer.

**Methods:**

Fluorescence quantitative PCR and immunohistochemistry were used to detect the expression of the LIMK1 gene in cancer and adjacent tissues of 20 clinical colon cancer samples. The overexpression plasmids of LIMK1 and STK25 were constructed. An shRNA specific to LIMK1 was synthesized and transfected into colon cancer cell lines. The expression levels of EMT-related markers in cell lines were detected by real-time PCR. The effects of LIMK1 and STK25 on the proliferation and invasion of colon cancer cell lines were detected by CCK-8 assay, Transwell, and clonogenesis.

**Results:**

LIMK1 interacted with STK25 and was highly expressed in colon cancer. High expression of LIMK1 and STK25 is associated with poor prognosis in colon cancer patients. LIMK silencing inhibits proliferation, invasion, and EMT of colon cancer. Cotransfection of LIMK1 and STK25 promotes the malignant progression and EMT of colon cancer.

**Conclusion:**

Protein interaction between LIMK1 and STK25 occurs. Overexpression of LIMK1 and STK25 plays a role in promoting cell proliferation and invasion in colon cancer tissues and cells. They also play a role in promoting the occurrence and development of colon cancer.

## 1. Introduction

Colorectal cancer is one of the most common gastrointestinal tumors [1, 2]. According to the American Cancer Society (ACS), the new incidence of colorectal cancer ranks third among malignant tumors. Its mortality rate ranks second among all kinds of malignant tumors [1]. The incidence rate of colorectal cancer in developed countries is higher than that in underdeveloped countries [3]. In recent years, the incidence and mortality of colon cancer have increased significantly [4]. The occurrence and development of colorectal cancer and the mutation of the tumor suppressor gene inside the body and expression decrease have big concern [5]. Changes in the expression levels of these genes affect tumor signaling pathways, such as the Wnt/*β*-catenin signaling pathway and the epithelial-mesenchymal (EMT) process [6–8].

EMT plays an important role in the metastasis and invasion of malignant tumors [9]. During EMT, some important skeletal structures of epithelial cells will undergo great changes, such as E-cadherin and Vimentin [10]. The reduction or deletion of E-cadherin is considered to be an important marker of EMT in tumor cells [11]. This change is considered to be a potential mechanism for distant metastasis and invasion of tumor cells [12].

LIMK1 is one of the two members of the LIM kinase (LIMK) protein family [13]. The physiological function of LIMK1 is mainly to phosphorylate Cofilin to become inactivated p-Cofilin and then participate in the reorganization of the cell actin skeleton [14]. There are many mechanisms regulating LIMKl activation in vivo. Activated LIMKl acts as a bridge. LIMK1 is a link between extracellular stimulation and cytoskeletal stability [15]. In recent years, the significance of LIMK1 in tumorigenesis has attracted extensive attention [16]. Studies have found that LIMK1 plays an important role in the genesis and development of human breast cancer cells [15]. LIMK1 is overexpressed in prostate tumors and prostate cancer cell lines [17]. Metastatic prostate cancer cells also have high concentrations of phosphorylated Cofilin. LIMK1 plays an important role in the regulation of tumor cell division and invasiveness [18]. LIMK1 may be one of the key molecules that cause tumor cell invasion and metastasis [19, 20]. However, the expression of LIMK1 in colon cancer and its molecular mechanism on the proliferation and migration of colon cancer cells remain to be further studied.

Germinal center kinase (GCK) is a family of conserved silk/threonine protein kinases [21]. In the same family as the P21-activated protein kinase (PAK) family, the STE20-like kinases are involved in a wide range of biological effects [22]. Some members can produce corresponding stress responses to external stimuli, such as cell proliferation, cell death, and cytoskeleton rearrangement. STK25, as an important member of GCK III, participates in the above basic physiological processes [23]. At the same time, STK25 plays an important role in energy metabolism and tumor genesis and development [24, 25].

This study analyzed the expression and biological role of LIMK1 and STK25 in colon cancer. The effect of LIMK1 and STK25 on the malignant progression of colon cancer was analyzed.

## 2. Methods

### 2.1. Cell Culture

Colon cancer cells were cultured in a DMEM complete medium containing 10% fetal bovine serum (penicillin 1 × 10^5^U/L and streptomycin 100 mg/L). The cultured cells were placed in a 37°C saturated humidity incubator (containing 5% CO_2_). The media were changed once every 2 days. When the degree of integration reached about 90%, the passage was passed.

### 2.2. Cell Transfection

The transfection complex shRNA-lipofectamine was prepared. The LIMK1 interfering plasmid shRNA and control plasmid shRNA-NC were diluted with 100 *μ*L serum-free medium and mixed to obtain solution A.7.5 *μ*L Lipofectamine2000 was diluted in 100 *μ*L serum-free medium. After incubation at room temperature for 5 min, liquid B was obtained. The liquids of A and B were added together and mixed gently. The shRNA-lipofectamine complex was obtained after incubation at room temperature for 20 min. The cell concentration was adjusted to 1 × 10^5^/mL and the cells were inoculated in 6-well plates with 2 mL in each well. The cells were cultured in the incubator for 24 h. When the cells covered about 80% of the surface area, the supernatant was discarded. The cells were cleaned with precooled PBS. 200 *μ*L shRNA LIMK1 or shRNA-NC transfection complex was added to each well, respectively. After 4 h culture in the incubator, the supernatant was discarded. 3 mL of complete culture medium was added and cultured in the incubator for 48 h to obtain cells, respectively. After receiving the samples, relevant tests were carried out.

### 2.3. CCK 8 Experiments

Logarithmic colon cancer cells were digested and inoculated into 96-well plates at a rate of 5000 per well. The cells were divided into groups and continued to be cultured after the cells adhered to the wall overnight. The medium was replaced with a fresh medium at 72 h. 10 *μ*L of CCK-8 solution was obtained and it was added to the test well. It was incubated in an incubator for 1 h. The wavelength parameter of the microplate reader was set to 450 nm. The optical density value of each hole was detected (D (450)).

### 2.4. Transwell Experiment

After thawing the matrix glue, the matrix glue was diluted with the medium according to 1 : 7 ratio. A pipette was used to drain 100 *μ*L and then it was added to the chamber. It was incubated at 37°C for 2 h for solidification. Logarithmic growth cells were taken and diluted into 5 × 10^5^/mL cell suspension with the medium. 200 *μ*L was added to the chamber. 750 *μ*L of the medium containing 10% FBS was added to the well outside the chamber. After 48 h culture in the cell incubator, the cells were removed. The liquid in the inner chamber was absorbed and fixed with 5% formaldehyde for 10 min. 1 mL of crystal violet (5%) was obtained and added into the holes of the 24-well plate. It was placed in a small chamber and stained for 20 min. After dyeing, it was washed with PBS and the walls of the chamber were gently wiped before air drying. Five fields (×400) were randomly selected under the microscope. ImageJ software was used to count the number of penetrating cells.

### 2.5. Clone Formation Experiment

LipofectamineTM2000 was transfected into the cells. Trypsin digests cells. Blow and mix to form single cell suspension and inoculate in a Petri dish. Petri dishes were placed in 37°C, 5% CO_2_ constant temperature incubator for continuous 2 weeks. The formation of cell clones was observed under a microscope. When the single cell clones were grown, the culture was drained. 1 × PBS 5 ml was cleaned twice. Coomassie bright blue R-250 staining was added for 2 h. It was placed on a shaker to wash and stain until the background is clean. ImageJ software was used to set the standard size of cell cloning. The number of clones above standard size was counted in each dish.

### 2.6. Subcutaneous Graft Tumor Model

Female nude mice aged 4–6 weeks were purchased from the Shanghai Institute of Zoology and kept in an SPF animal house. Nude mice were fed adaptively for 1 week. They were randomly divided into sh-NC and sh-LIMK1 groups, with 6 rats in each group. The cells at the logarithmic growth stage were digested with 0.25% trypsin, and the cells were collected and the concentration was adjusted to 1 × 10^7^ cells/mL. The nude mice in the two groups were subcutaneously injected with 0.1 mL corresponding tumor cell suspension. The diameter and weight of tumors were measured daily and analyzed statistically. On day 28, all nude mice were euthanized. Subcutaneous tumor tissue was isolated. Mice were weighed and then the measurements were recorded. Tumor inhibition rate = ((mean tumor mass of control group − mean tumor mass of treatment group) ÷ mean tumor mass of control group) × 100%. Animal experiments are approved by the Ethics Committee of the First Affiliated Hospital of Wenzhou Medical University.

### 2.7. qRT-PCR

The cell concentration was adjusted to 1 × 10^5^/mL and the cells were inoculated in a 6-well plate with 2 mL per well. It was cultivated for 24 h in a cell incubator. The cells were collected by trypsinization and centrifugation. The total RNA was extracted with a TRIzol reagent. Refer to Prime Script 1st Strand cDNA Synthesis Kit for reverse transcription kit instructions to synthesize cDNA. Refer to the instructions for the SYBR Premix Ex Taq TM real-time fluorescent quantitative PCR kit. The primers were designed and synthesized by Shanghai Sangon Biotech Company (Shanghai, China): GAPDH (human)-forward sequence 5′-AGAAGGCTGGGGCTCATTTG-3′GAPDH (human)-reverse sequence 5′-AGGGGCCATCCACAGTCTTC-3′. The 2^−△△CT^ method was used to analyze the expression differences of the target gene among the groups.

### 2.8. Western Blot

After the cells were treated, total protein was extracted according to the instructions. After extraction, protein concentration was measured with a BCA kit. After thermal denaturation, 12% SDS-PAGE was performed. The sample size of protein in each well was 40 *μ*g. The film was transferred with 250 mA constant current. It was sealed with 5% skim milk powder at room temperature for 3 h. The primary antibodies and monoclonal antibodies LIMK1 and STK25 (volume dilution ratio 1 : 1000) were incubated at 4°C overnight. They were washed three times with TBST, 10 min each. Secondary antibodies were incubated at room temperature for 2 h on a shaker (dilution ratio: 1 : 2000). They were washed three times with TBST, 10 min each. ECL chemiluminescence development was observed.

### 2.9. Immunofluorescence Colocation

The cell concentration was adjusted to 1 × 10^5^/mL and the cells were inoculated in 6-well plates with 2 mL in each well. The cells were cultured in the incubator for 24 h, rinsed with precooled PBS twice, fixed with 4% paraformaldehyde for 15 min, treated with 0.1% Triton at room temperature for 15 min, and washed with PBS twice. 5% FBS was sealed for 30 min. Primary antibodies of LIMK1 and STK25 were incubated at 4°C overnight. The secondary antibody was added after PBS cleaning. The cells were incubated at 37°C for 1 h, and DAPI was incubated at room temperature under dark conditions for 15 min for contrast staining. Photographs were taken under a fluorescence microscope.

### 2.10. Statistical Analysis

Data were expressed as mean ± standard deviation (mean ± SD). Data were compared with SPSS 17.0 statistical software. The comparison between the two groups was performed by *t* test. Multivariate comparisons were performed using one-way ANOVA. Pearson correlation analysis was used to determine coexpression correlation. *P* < 0.05 was considered a statistically significant difference.

## 3. Results

### 3.1. LIMK1 Is Highly Expressed in Colon Cancer and Is Associated with Poor Prognosis

**Figure 1:**
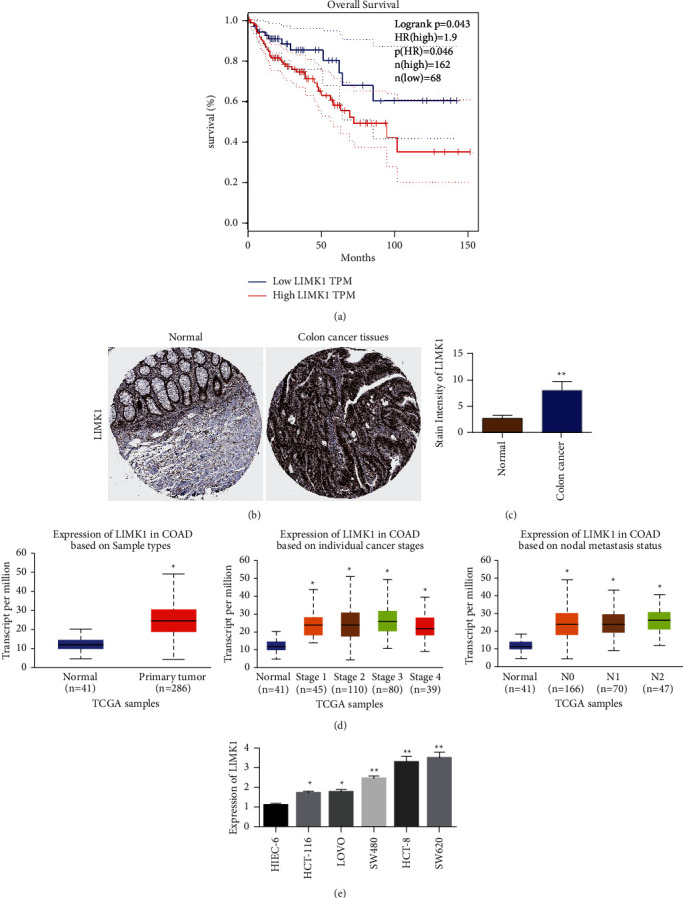
LIMK1 is upregulated in colon cancer and is associated with poor prognosis of colon cancer. (a) Survival analysis of colon cancer patients with different expression levels of LIMK1. (b) Immunohistochemical test results of LIMK1 in normal colon tissue and colon cancer tissue. The immunohistochemical images were obtained from The Human Protein Atlas website. (c) Immunohistochemical statistical results of LIMK1 in normal colon tissue and colon cancer tissue. (d) UALCAN website analyzes the relationship between LIMK1 expression and colorectal cancer staging and metastasis. (e) The expression level of LIMK1 in normal intestinal epithelial cells and colorectal cancer cells. ^*∗*^*p* < 0.05; ^*∗∗*^*p* < 0.01.

In order to study the role of LIMK1, the correlation between LIMK1 expression level and prognosis of colon cancer patients was analyzed by using TCGA data. Analysis results showed that patients with high LIMK1 expression (*n* = 162) had a shorter survival time compared with patients with low LIMK1 expression (*n* = 68) (Figure 1(a)). Immunohistochemical results showed that the positive rate of LIMK1 in colon cancer tissues was higher than that in para-cancer tissues (Figure 1(b)). TCGA database analysis results showed that the expression of LIMK1 was also related to the staging and metastasis of colon cancer patients (Figure 1(c)). Real-time quantitative PCR results showed that the expression of LIMK1 in 20 colon cancer tissues was higher than that in adjacent tissues (Figure 1(d)). Compared with intestinal epithelial hiEC-6, LIMK1 is highly expressed in colon cancer cell lines. Moreover, SW620 and HCT-8 cells had the highest expression level (Figure 1(e)).

### 3.2. Influence of Interfering LIMK1 Expression on EMT

**Figure 2:**
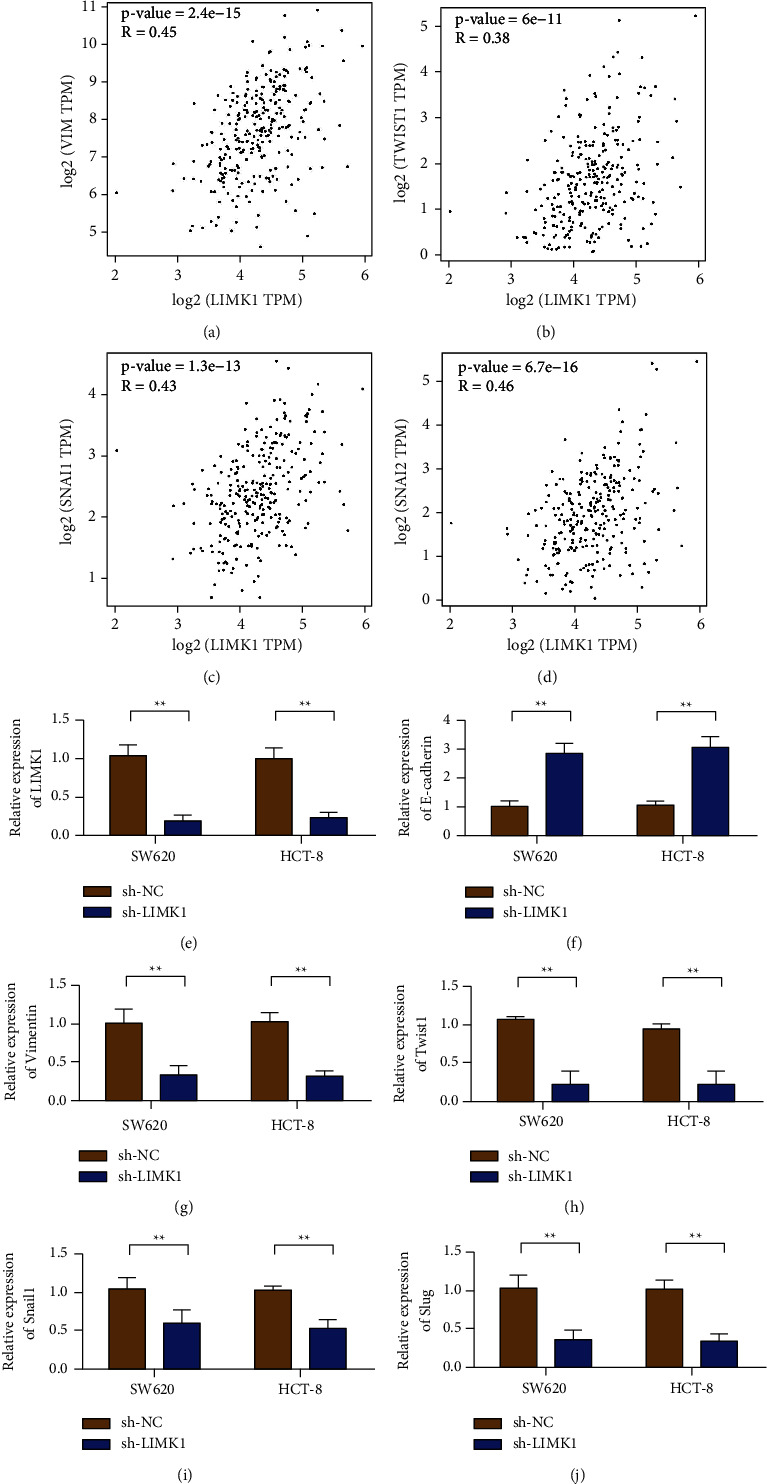
LIMK1 regulates the expression of EMT markers. (a) The expression level of LIMK1 is positively correlated with the coexpression of Vimentin. (b) The expression level of LIMK1 is positively correlated with the coexpression of Twist1. (c) The expression level of LIMK1 is positively correlated with the coexpression of Snail. (d) The expression level of LIMK1 is negatively correlated with the coexpression of Slug. (e) The efficiency of knockdown of LIMK1 in SW620 and HCT-8 cells was verified. (f) After knocking down LIMK1 in SW620 and HCT-8 cells, the expression of E-cadherin was detected. (g) After knocking down LIMK1 in SW620 and HCT-8 cells, the expression of Vimentin was detected. (h) After knocking down LIMK1 in SW620 and HCT-8 cells, the expression of Twist1 was detected. (i) Detection of Snail1 expression level after knocking down LIMK1 in SW620 and HCT-8 cells. (j) After knocking down LIMK1 in SW620 and HCT-8 cells, Slug expression was detected. ^*∗∗*^*p* < 0.01.

Coexpression correlation analysis results showed that in colon cancer tissues, the coexpression of LIMK1 was positively correlated with EMT marker Vimentin (Figure 2(a)). In addition, LIMK1 is positively coexpressed with transcription factors Twist1, Snail1, and Slug (Figures 2(b)–2(d)). The expression of LIMK1 in SW620 and HCT-8 cells transfected with shRNA LIMK1 was detected by qRT-PCR. The results showed that, compared with the control group (shRNA-NC), the expression of LIMK1 in the transfected group was decreased (Figure 2(e)). qRT-PCR results showed that, after LIMK1 silencing, the expression of E-cadherin was increased (Figure 2(f)), while the expression of Vimentin was decreased (Figure 2(g)). The expression levels of transcription factors Twist1, Snail1, and Slug also decreased after LIMK1 was knocked out (Figures 2(h)–2(j)).

### 3.3. Interference of LIMK1 Expression Inhibits the Proliferation and Invasion of Colon Cancer Cells

**Figure 3:**
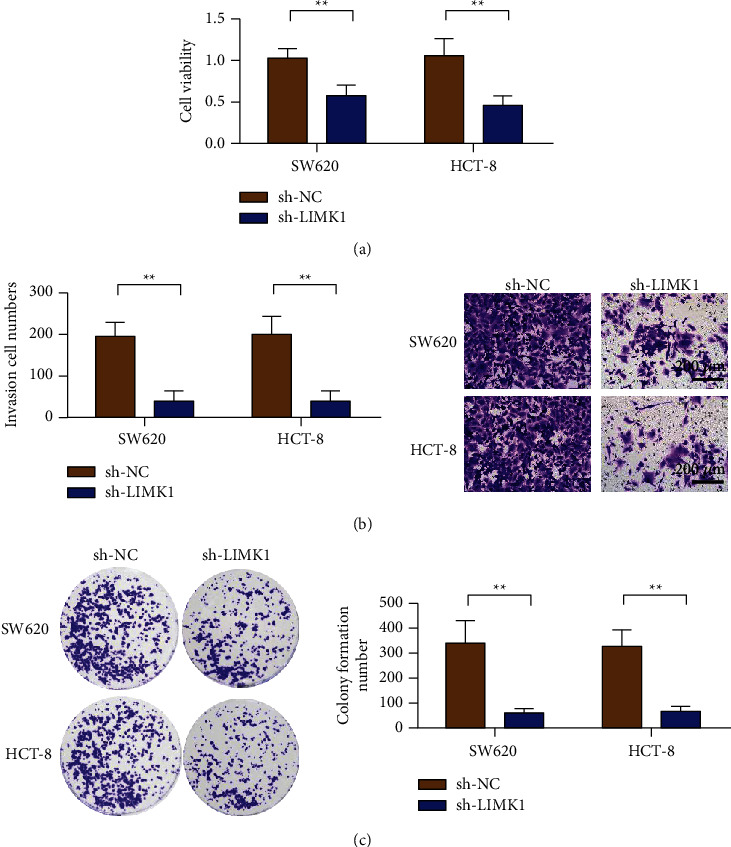
Knockdown of LIMK1 inhibits colon cancer proliferation, invasion, and colonization. (a) Knockdown of LIMK1 inhibits cell viability detection of colon cancer cells SW620 and HCT-8. (b) Knockdown of LIMK1 inhibits the invasion of colon cancer cells SW620 and HCT-8 (magnification: 100x). (c) Knockdown of LIMK1 inhibits the clonogenic ability of colon cancer cells SW620 and HCT-8. ^*∗∗*^*p* < 0.01.

CCK-8 results showed that cell activity was decreased in the shRNA-LIMK1 group compared with the control group (shRNA-NC) (Figure 3(a)). Transwell results showed that, compared with the control group (shRNA-NC), the number of colon cancer cells crossing the basement membrane was significantly reduced in the shRNA-LIMK1 group, with a statistically significant difference (Figure 3(b)). Compared with the control group (shRNA-NC), the number of colon cancer cell clones in the shRNA-LIMK1 treatment group was significantly reduced, with a statistically significant difference (Figure 3(c)).

### 3.4. In Vivo Experiment to Verify the Effect of LIMK1 Gene Silencing on Colon Cancer Cell Proliferation and EMT

**Figure 4:**
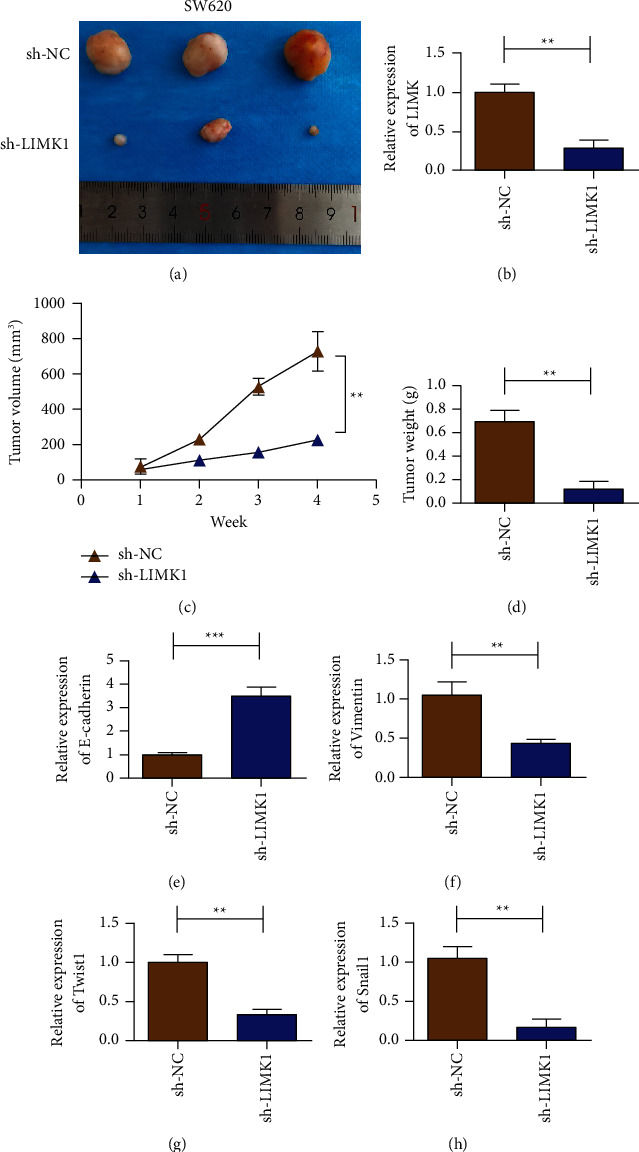
LIMK1 gene knockdown inhibits the growth of colon cancer tumors. (a) Representative pictures of tumors in the control group and LIMK1 knockdown group. (b) Detection of the expression of LIMK1 in the tumor tissue after SW620 cells transfected with sh-LIMK1 in tumor-bearing mice. (c) Detection of the tumor volume of SW620 cells transfected with sh-LIMK1. (d) Detection of the tumor weight of SW620 cells transfected with sh-LIMK1. (e) Detection of the expression of E-cadherin in tumor tissue after SW620 cells transfected with sh-LIMK1 in tumor-bearing mice. (f) Detection of the expression of Vimentin in tumor tissue after SW620 cells transfected with sh-LIMK1 in tumor-bearing mice. (g) Detection of the expression of Twist1 in the tumor tissue after SW620 cells transfected with sh-LIMK1 in tumor-bearing mice. (h) Detection of the expression of Snail1 in the tumor tissue after SW620 cells transfected with sh-LIMK1 in tumor-bearing mice. ^*∗∗*^*p* < 0.01, ^*∗∗∗*^*p* < 0.001.

SW620 cells were selected to study the effect of the LIMK1 gene on proliferation and EMT in colon cancer cells. Animal experimental results showed that silencing LIMK1 could reduce tumor size (Figure 4(a)). Real-time quantitative PCR results showed that the expression of LIMK1 in the sh-LIMK1 group was lower than that in the corresponding sh-NC group (Figure 4(b)). Meanwhile, the results of the growth curve showed that the growth rate of colon cancer cells was significantly reduced after LIMK1 gene silencing compared with the control group (Figure 4(c), *P* < 0.01). Silencing LIMK1 also reduced tumor weight (Figure 4(d)). Results of E-cadherin expression detection showed that, after LIMK1 silencing, the expression of E-cadherin in tumor tissues increased (Figure 4(e)), while that of Vimentin decreased (Figure 4(f)). Transcription factors Twist1 and Snail1 expression detection results showed that Twist1 and Snail1 expression decreased in tumor tissues after LIMK1 silencing (Figures 4(g)–4(h)).

### 3.5. LIMK1 Interacts with STK25

**Figure 5:**
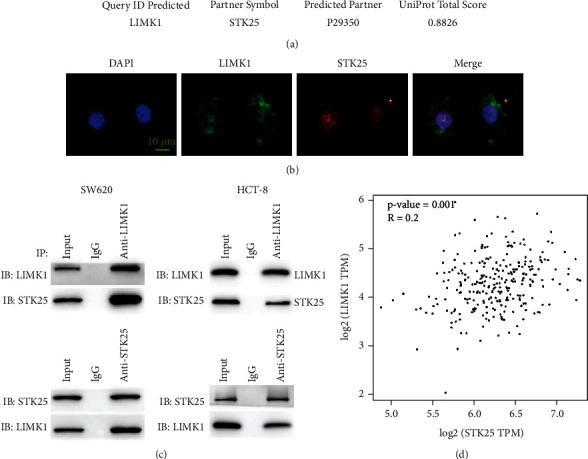
Protein-protein interaction between LIMK1 and STK25. (a) LIMK1 interaction protein prediction. (b) Immunofluorescence colocalization of LIMK1 and STK25 in colon cancer cells (scale bar: 10 *μ*m). (c) Co-IP experiment verifies the interaction between LIMK1 and STK25. (d) Co-expression of LIMK1 and STK25 in colon cancer tissue is positively correlated.

We predict LIMK1 protein interactions through the FpClass website (http://dcv.uhnres.utoronto.ca/FPCLASS/ppis/). The results showed that STK25 may interact with LIMK1 (Figure 5(a)). Immunofluorescence staining results showed that LIMK1 and STK25 were colocated in the cytoplasm (Figure 5(b)). Co-IP experiment results further confirmed the interaction between LIMK1 and STK25 (Figure 5(c)). Coexpression correlation analysis results showed that LIMK1 and STK25 had a positive coexpression correlation in colon cancer tumor tissues (Figure 5(d)).

### 3.6. STK25 Promoted the Proliferation and Invasion of Colon Cancer SW620 and HCT-8 Cells In Vitro

**Figure 6:**
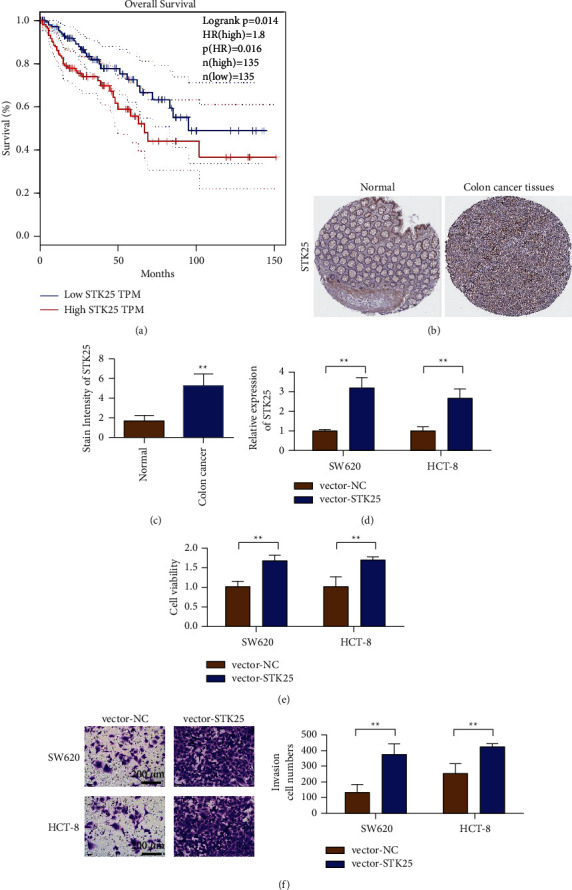
STK25 is upregulated in colon cancer and is associated with poor prognosis of colon cancer. (a) Survival analysis of colon cancer patients with different STK25 expression levels. (b) Immunohistochemical test results of STK25 in normal colon tissue and colon cancer tissue. The experimental results were obtained from The Human Protein Atlas website. (c) The statistical results of immunohistochemical detection of STK25 in normal colon tissue and colon cancer tissue. (d) RT-qPCR analysis of STK25 expression in CRC tissues. (e) Detection of transfection efficiency of overexpression of STK25 in SW620 and HCT-8 cells. (f) Overexpression of STK25 promotes the detection of cell viability of colon cancer cells SW620 and HCT-8 (magnification: 100x). (g) Overexpression of STK25 promotes the invasion of colon cancer cells SW620 and HCT-8. ^*∗∗*^*p* < 0.01.

The correlation between STK25 expression level and prognosis of colon cancer patients was analyzed by TCGA data. Analysis results showed that compared with patients with low STK25 expression (*n* = 135), patients with high STK25 expression (*n* = 135) had a shorter survival time (Figure 6(a)). Immunohistochemical results showed that the positive rate of STK25 in colon cancer tissues was higher than that in para-cancer tissues. Furthermore, STK25 was mainly expressed in the cytoplasm of colon cancer tissues, which was significantly stronger than that of para-cancer tissues (Figures 6(b) and 6(c)). Detection results of STK25 expression level showed that the overexpressed plasmid could upregulate the expression level of STK25 in SW620 and HCT-8 cells (Figure 6(d)). CCK-8 results showed that, compared with the control group, the cell activity of the SKT25 overexpressed group was enhanced (Figure 6(e)). These results suggest that SKT25 can enhance the cell activity of colon cancer SW620 and HCT-8 cells. The results of the Transwell in vitro invasion experiment showed that, compared with the control group, the number of invasive cells in the SKT25 overexpression group was significantly increased (Figure 6(f)). These results suggest that SKT25 can significantly enhance the invisibility of colon cancer SW620 and HCT-8 cells in vitro.

### 3.7. The Interaction between LIMK1 and STK25 Promotes the Malignant Progression of Colon Cancer

**Figure 7:**
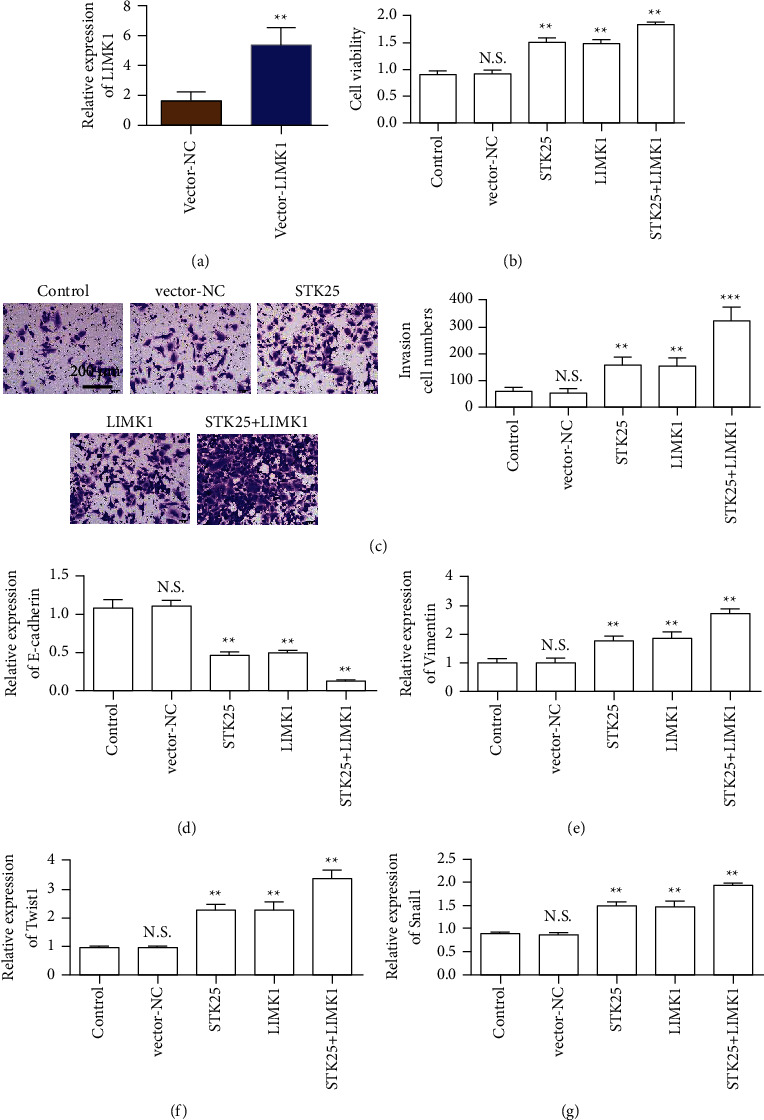
Overexpression of STK25 increases the tumor-promoting effect of LIMK1. (a) Detection of LIMK1 expression in SW620 cells. (b) SW620 cell proliferation rate detection. (c) SW620 cells are tested by Transwell (magnification: 100x). (d) Detection of E-cadherin expression in SW620 cells. (e) Detection of Vimentin expression in SW620 cells. (f) Detection of Twist1 expression in SW620 cells. (g) Detection of Snail1 expression in SW620 cells.

The detection results of LIMK1 expression showed that the overexpressed plasmid upregulated the expression of LIMK1 in SW620 and HCT-8 cells (Figure 7(a)). The results of the CCK-8 assay showed that the cell activity of the LIMK1 and SKT25 overexpressed groups was enhanced compared with the control group. Meanwhile, cells cotransfected with LIMK1 and STK25 showed the highest activity (Figure 7(b)). The results of the Transwell in vitro invasion assay showed that the number of invasive cells in the group overexpressing LIMK1 and STK25 alone was significantly higher than that in the control group. The number of invaded cells in the simultaneous cotransfection group of LIMK1 and STK25 was the largest (Figure 7(c)). E-cadherin expression detection results showed that the expression of E-cadherin was decreased in LIMK1 and STK25 overexpression groups alone. The expression level of the LIMK1 and STK25 cotransfected groups was the lowest (Figure 7(d)). The expression of Vimentin increased after overexpression of LIMK1 and STK25 alone. The expression level of Vimentin in LIMK1 and STK25 cotransfected group was the highest (Figure 7(e)). The expression levels of transcription factors Twist1 and Snail1 were detected and the expression levels of Twist1 and Snail1 increased after LIMK1 and STK25 were overexpressed alone. The expression levels of Twist1 and Snail1 in LIMK1 and STK25 cotransfected groups were the highest (Figures 7(f) and 7(g)).

## 4. Discussion

Colorectal cancer is one of the most common malignant tumors [26]. With the improvement of people's living standards and the change of diet structure, the incidence rate has been increasing year by year in recent years [27]. Although current early screening for colorectal cancer has reduced mortality, there are many missed diagnoses. Tumors are often found in the middle and late stages [28]. In addition, although advances in the diagnosis and treatment of colorectal cancer have improved 5-year survival, tumor proliferation and metastasis are still a challenge in the treatment of colorectal cancer [29]. In order to cope with advanced colorectal cancer, inhibit tumor metastasis, and prevent tumor spread and recurrence, effective targeted intervention, combined with traditional surgery, chemotherapy, and radiotherapy, is expected to become an important means to prolong the survival of patients with colorectal cancer [30]. EMT initiates metastasis, which plays a key role in the poor prognosis of tumors [9].

Metastasis of tumor cells is a continuous process regulated by many factors and developed in many stages [31]. In this process, all factors that can affect the proliferation rate and metastasis potential of tumor cells are crucial to the eventual formation of tumor metastasis [32]. The ability of tumor cells to proliferate and migrate is the most basic condition [33]. At present, a large number of studies have confirmed that LIM kinase (LIMK) is highly expressed in a variety of human tumors, especially in highly aggressive tumors [34]. LIMK regulates actin polymerization by phosphorylation and inactivation of actin depolymerization factor Cofilin [35]. Therefore, LIMK is involved in regulating various biological behaviors of tumor cells, including tumor angiogenesis, tumor cell migration and proliferation, and cell cycle progression, promoting tumor invasion and metastasis [36]. Therefore, LIMK may be a potential therapeutic target to inhibit tumor invasion and metastasis [37]. Studies have shown that high expression of LIMK1 can promote invasion and metastasis of osteosarcoma cells resistant to vincristine. The migration of osteosarcoma cells decreased after LIMK1 silencing [38]. LIMK1 can enhance cell migration and promote tumor cell metastasis. Knockout of LIMK1 inhibits migration and invasion of hepatocellular carcinoma cells from rat peritoneal effusion [39]. The inhibition of LIMK effectively blocks TGF-*β*-induced migration and invasion by reducing cell migration [40]. The results of this study showed that knockdown of LIMK1 inhibited the proliferation of colon cancer cells. At the same time, the invasion and clonogenesis of tumor cells were also reduced after LIMK1 knockdown. Further in vivo studies showed that colon cancer cells with LIMK1 knockdown grew slower in a nude mouse model of colon cancer. Meanwhile, the expression of E-cadherin increased after LIMK1 knockdown, while the expression of Vimentin, Twist1, and snail1 decreased. It was further confirmed that reduced LIMK1 expression could inhibit the proliferation and EMT of colon cancer cells. This study also found that E-cadherin expression was increased after LIMK1 knockdown at the cellular level. Vimentin is less expressed. These results suggest that knocking down LIMK1 can inhibit EMT of colon cancer SW620 and HCT-8 cells and reduce invasion and migration.

STK25 is widely expressed in mouse, rat, and human tissues and organs [41]. Under normal conditions, STK25 locates in the Golgi apparatus of cells and works in the signaling cascade required for cell migration [42]. Studies have shown that altering the activity or the expression level of STK25 and regulating cell proliferation/apoptosis/polarity by STK25/GM130/PDCD10 may provide new directions for tumor therapy [43–46]. Transwell assay showed that knockdown of STK25 inhibited the migration of colon cancer cells. This study detected a high expression of STK25 in colon cancer. Compared with the control group, the expression of E-cadherin protein was significantly increased and the expression of Vimentin was decreased after STK25 knockdown. These results indicated that STK25 knockdown inhibited the EMT process and further inhibited the distant metastasis and diffusion of colon cancer cells. These results also suggest that STK25 plays a key role in the development and metastasis of colon cancer. In this study, LIMK1 can interact with STK25 to promote the EMT process in colon cancer. The overexpressed plasmid group cotransfected with LIMK1 and STK25 showed the strongest proliferation and invasion ability of colon cancer cells.

The limitation of this study is that the specific molecular mechanism of the LIMK1 gene in the occurrence and development of colon cancer is still unclear. It remains to be further studied how the gene promotes the proliferation and invasion of cancer cells through the cellular signaling pathway. Another limitation is lack of continuous follow-up of clinical study subjects. To reveal the effect of LIMK1 expression on prognosis, further clinical studies should be supplemented and improved.

## 5. Conclusion

LIMK1 can mediate the proliferation, invasion, migration, and EMT of colon cancer cells through its interaction with STK25. This study provides a new understanding of the mechanism of LIMK1 leading to proliferation, invasion, migration, and EMT of colon cancer cells. The LIMK1 and STK25 pathways may be new therapeutic targets for colon cancer. This study provides new ideas for clinical treatment and drug development of colon cancer.

## Data Availability

The analyzed datasets generated during the study are available from the corresponding author on reasonable request.
